# Combination of Defined CatWalk Gait Parameters for Predictive Locomotion Recovery in Experimental Spinal Cord Injury Rat Models

**DOI:** 10.1523/ENEURO.0497-20.2021

**Published:** 2021-03-05

**Authors:** Ivanna K. Timotius, Lara Bieler, Sebastien Couillard-Despres, Beatrice Sandner, Daniel Garcia-Ovejero, Florencia Labombarda, Veronica Estrada, Hans W. Müller, Jürgen Winkler, Jochen Klucken, Bjoern Eskofier, Norbert Weidner, Radhika Puttagunta

**Affiliations:** 1 Machine Learning and Data Analytics Lab, Department of Computer Science, Friedrich-Alexander-Universität Erlangen-Nürnberg, Erlangen 91052, Germany; 2 Department of Electronics Engineering, Satya Wacana Christian University, Salatiga 50711, Indonesia; 3 Institute of Experimental Neuroregeneration, Paracelsus Medical University, Salzburg 5020, Austria; 4 Spinal Cord Injury and Tissue Regeneration Center Salzburg (SCI-TReCS), Paracelsus Medical University, Salzburg 5020, Austria; 5 Austrian Cluster for Tissue Regeneration, Vienna 1200, Austria; 6 Spinal Cord Injury Center, Heidelberg University Hospital, Heidelberg 69118, Germany; 7 Laboratory of Neuroinflammation, Hospital Nacional de Paraplejicos, Servicio de Salud de Castilla-La Mancha, Toledo 45071, Spain; 8 Laboratorio de Bioquímica Neuroendocrina, Instituto de Biología y Medicina Experimental, Consejo Nacional de Investigaciones Científicas y Técnicas, Buenos Aires, C1428 ADN, Argentina; 9 Departamento de Bioquímica Humana, Facultad de Medicina, Universidad de Buenos Aires, C1121A6B, Buenos Aires, Argentina; 10 Molecular Neurobiology Lab, Department of Neurology, University of Düsseldorf, Düsseldorf 40225, Germany; 11 Department of Molecular Neurology, University Hospital Erlangen, University of Erlangen-Nürnberg, Erlangen 91054, Germany

**Keywords:** CatWalk, gait parameter, linear discriminant analysis, locomotion recovery, preclinical development, spinal cord injury

## Abstract

In many preclinical spinal cord injury (SCI) studies, assessment of locomotion recovery is key to understanding the effectiveness of the experimental intervention. In such rat SCI studies, the most basic locomotor recovery scoring system is a subjective observation of animals freely roaming in an open field, the Basso Beattie Bresnahan (BBB) score. In comparison, CatWalk is an automated gait analysis system, providing further parameter specifications. Although together the CatWalk parameters encompass gait, studies consistently report single parameters, which differ in significance from other behavioral assessments. Therefore, we believe no single parameter produced by the CatWalk can represent the fully-coordinated motion of gait. Typically, other locomotor assessments, such as the BBB score, combine several locomotor characteristics into a representative score. For this reason, we ranked the most distinctive CatWalk parameters between uninjured and SC injured rats. Subsequently, we combined nine of the topmost parameters into an SCI gait index score based on linear discriminant analysis (LDA). The resulting combination was applied to assess gait recovery in SCI experiments comprising of three thoracic contusions, a thoracic dorsal hemisection, and a cervical dorsal column lesion model. For thoracic lesions, our unbiased machine learning model revealed gait differences in lesion type and severity. In some instances, our LDA was found to be more sensitive in differentiating recovery than the BBB score alone. We believe the newly developed gait parameter combination presented here should be used in CatWalk gait recovery work with preclinical thoracic rat SCI models.

## Visual Abstract

**Figure:**
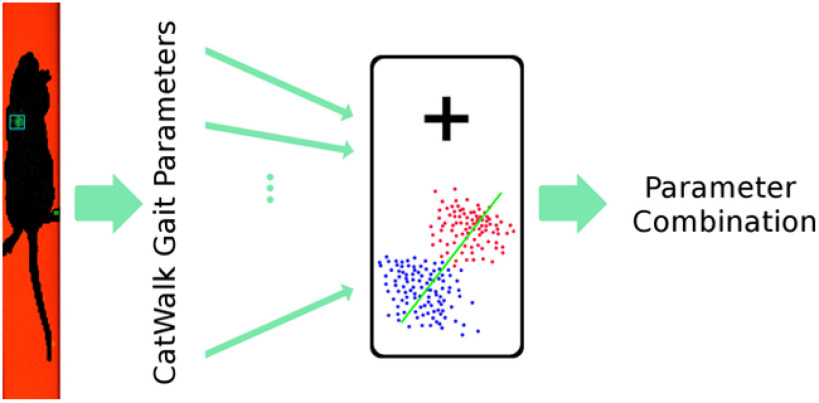

## Significance Statement

As a quantitative locomotion analysis system, CatWalk provides an objective assessment of gait for rodents by computing numerous parameters. This gives an alternative to the current popular subjective locomotor assessment for rat spinal cord injury (SCI) models, i.e., Basso Beattie Bresnahan (BBB) score. As SCI affects multiple gait parameters, analyzing gait is challenging. Here, we developed a CatWalk gait parameter combination for sensitive and efficient gait recovery assessment in rat thoracic SCI models.

## Introduction

Following a spinal cord injury (SCI), disruption to descending fine and gross motor control, as well as to ascending sensory input, leads to functional deficits, all the way up to paralysis. Standardized and consistent behavioral assessments are required to accurately monitor any spontaneous or induced recovery that follows.

Preclinical rat SCI studies used in developing treatments for SCI (Cheriyan et al., 2014; Silva et al., 2014; Kjell and Olson, 2016; Ahuja et al., 2017) commonly rely on a motor assessment based on a standardized scale involving the observation of various characteristics to generate a non-parametric score (Bhimani et al., 2017). For rat thoracic contusion SCI models, in particular, Basso et al. (1995) developed a standard non-parametric locomotion assessment named Basso Beattie Bresnahan (BBB) locomotion scale. This subjective locomotion assessment scores hindlimb (HL) movement, joint movement, weight supported plantar stepping, forelimb (FL)–HL coordination, toe clearance, trunk stability, and tail placement in an open field test setting. In the BBB locomotion assessment, the examiners’ experience attributes a significant aspect of the score’s reliability (Basso et al., 1996).

**Figure 1.**
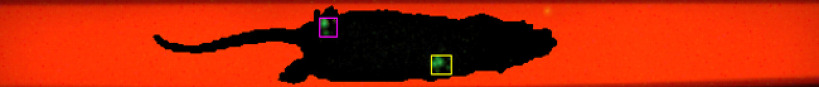
An example of CatWalk image captured from below an injured rat. The paws in contact with the glass walkway are indicated with the colored boxes (magenta: right hindpaw; yellow: left forepaw).

CatWalk (Noldus Information Technology), on the other hand, is a locomotion analysis system specifically assessing gait performance based on rodent footsteps and body silhouette. Introduced by Hamers, CatWalk was initially designed to assess FL–HL coordination (Hamers et al., 2001, 2006). Using this system, paw positions during walking can be recorded using a high-speed camera positioned beneath the glass walkway (Fig. 1). Based on the recorded video and paw positions, the CatWalk system computes a multitude of static and dynamic gait parameters, such as the base of support (BOS), stride length, swing speed, body speed, and regularity index (RI), to name a few. For example, the RI (Koopmans et al., 2005) is a gait parameter used to quantify FL–HL coordination based on the percentage of the defined normal step sequence patterns (NSSPs). The CatWalk apparatus provides further information than the traditional analysis of inked footprints collected on paper rolls, which can only deliver static gait parameters.

Since SCI affects several motor and sensory pathways, multiple gait parameters need to be analyzed simultaneously (Lakes and Allen, 2016). However, in the majority of published work, CatWalk parameters are evaluated individually (Garcia-Ovejero et al., 2014; Fagoe et al., 2016; Bhimani et al., 2017; Kappos et al., 2017; Slusarczyk et al., 2017; Bieler et al., 2018; Aceves et al., 2020; Heinzel et al., 2020). The large number of gait parameters as well as the variated gait effect of an injury often bring complications in the analysis of gait. Therefore, a combination of CatWalk gait parameters to faithfully characterize gait recovery in rat SCI models will be a valuable asset for future research studies.

Simple CatWalk parameter combinations have been used in a variety of disorders to assess gait, such as baseline parameter ratio (Datto et al., 2015, 2016; Hayakawa et al., 2015; Mondello et al., 2015), left-right-parameter ratio, left-right-parameter averaging, and subtraction (Liu et al., 2013; Parvathy and Masocha, 2013; Chen et al., 2014, 2017; Ishikawa et al., 2014; Muramatsu et al., 2014). In addition to these basic operations, researchers have used principal component analysis (PCA) on multiple CatWalk parameters to determine the most relevant parameters for locomotor phenotyping of mice (Zimprich et al., 2018). Also, linear regression of the Basso Mouse Scale (BMS) against each CatWalk parameter have been combined into a weighted score in thoracic mouse contusion SCI (Crowley et al., 2018), and classifiers developed based on high-dimensional CatWalk-derived data to classify gait pattern in Parkinson’s disease-relevant animal models and control groups (Fröhlich et al., 2018).

In contrast to this previous work, we propose a CatWalk parameter linear combination, which has been developed based on a linear discriminant analysis (LDA) of several gait parameters ranked according to the difference measured between uninjured and spinal cord injured rats. LDA was first introduced by Fisher (Fisher, 1936) as a method to determine a linear function that best discriminates two groups. Based on the most descriptive gait parameters identified, using LDA we were able to quantify gait recovery of different rat SCI models using a weighted linear combination of parameters. The designed LDA was then applied and validated to assess gait recovery in various SCI models, ranging from contusion, dorsal hemisection to dorsal column lesion. The gait recovery profile was reliably uncovered from most of these studies even when differences were not detectable by single CatWalk parameters or by the BBB score alone.

## Material and Methods

### Subjects and study protocols

In rats, the gait recovery assessment method was applied to three Th8/9 contusion SCI studies, one Th8/9 dorsal hemisection SCI, and one C4 bilateral dorsal column transection SCI, which are summarized in Table 1. The studies were conducted using two different versions of the CatWalk system, which have different camera frame rates (50 fps for CatWalk 7.1 and 100 fps for CatWalk XT).

**Table 1:** Overview of the studies

	Study 1	Study 2	Study 3	Study 4	Study 5
Animals	Female Fischer 344	Male Wistar	Female Fischer 344	Female Wistar	Female Fischer 344
SCI	Th8/9 Contusion Mod. and Mod.Sev (150 kDyn)	Th8 Contusion Mod.Sev. (200 kDyn)	Th8 Contusion Mod.Sev. (200 kDyn)	Th8/9 Dorsal Hemisection Scouten wire-knife transection	C4 bilateral dorsal column tungsten wire-knife transection
Time points of gait assessment	UI, 60 dpi	UI, 7, 30, 60 dpi	UI, 15, 22, 29, 36, 43 dpi	UI, 30, 60 dpi	UI, 30 dpi
CatWalk system	CatWalk XT	CatWalk 7.1	CatWalk XT	CatWalk XT	CatWalk XT
Groups	UI Veh.Mod Veh.Mod.-Sev Exp.Mod Exp.Mod.-Sev.	UI SCI+Veh. SCI+Prog.	Young SCI Old SCI	UI SCI Veh.	UI SCI Veh.

UI: uninjured; Prog.: progesterone; Mod.: Moderate; Sev.: severe; Veh.: vehicle; Exp.: experiment.

#### Study 1

This study was performed as published previously (Sandner et al., 2018) to examine the effects of Epothilone D (Epo D) on the functional recovery and regenerative potential following contusion SCI. For the Th8/9 contusion SCI model, adult female Fischer 344 rats (Charles River Deutschland GmbH, Envigo, Janvier Labs) weighing 160–180 g were used (10 weeks of age). Experiments were conducted in accordance with the European Union Directive (2010/63/EU) and institutional guidelines. Rats were fed *ad libitum*. The rats were anesthetized using a cocktail of ketamine (62.5 mg/kg; Medistar), xylazine (3.175 mg/kg; Bayer), and acepromazine (0.625 mg/kg; Sanofi-Ceva) in 0.9% sterile saline solution. The rats received a contusion SCI at midthoracic Th9 level (representing spinal level Th11) using the Infinite Horizon (IH) Impactor SCI device (Precision Systems and Instrumentation) with an impact force of 150 kilodynes (kDyn) without any additional dwell time. Two days postoperatively, twice a day, buprenorphine (0.03 mg/kg; Reckitt Benckiser) was given subcutaneously. Ampicillin (167 mg/kg; Ratiopharm, twice a day) was given subcutaneously as long as manual bladder evacuation was necessary. Rats received intraperitoneal injections of Epo D [Abcam, catalog #ab143616; 1.5 mg/kg body weight (bw)] dissolved in dimethylsulfoxide (DMSO; 3 mg/ml) and diluted 1:1 with prewarmed saline before injections of Epo D or vehicle (1:1 mixture of DMSO and saline, control group) on days 1 and 15 postinjury by a blinded unbiased experimenter. An animal in each cohort was excluded because of inadequate force impact curves on spinal cord contusion. Based on the variability in spinal cord displacement, rats were divided into two groups. A displacement value of 1000 μm allowed for equal distribution of animals into two cohorts (moderate: <1000 μm and moderate-severe: >1000 μm). These rats were then assigned to receive either vehicle or Epo D treatment. Rats were killed eight weeks postinjury.

##### Behavioral testing

At 60 d postinjury (dpi), CatWalk (CatWalk XT), and BBB tests were performed. The number of rats and CatWalk runs collected in this study are listed in Table 2. The BBB scores ranged from 10 to 19 at 60 dpi.

**Table 2:** Number of animals and CatWalk runs of Study 1

Study 1		#Animals	#Runs
Uninjured		6	27
Vehicle*	Mod.	8	31
	Mod-Sev.	7	30
Experiment*	Mod.	8	38
	Mod-Sev.	9	39

*60 dpi; Mod.: moderate; Mod-Sev.: moderate severe.

#### Study 2

This study was performed as published previously (Garcia-Ovejero et al., 2014) to examine the effects of progesterone on locomotor recovery and secondary damage following contusion SCI. Rats were handled in accordance with the guidelines published in the National Institutes of Health (NIH) Guide for the Care and Use of Laboratory Animals, the principles laid out in the Guidelines for the Use of Animals in Neuroscience Research published by the Society for Neuroscience, and European Union guidelines (Council Directive 86/609/EEC). Experimental procedures were approved by the Ethical Committee for Animal Welfare at the National Paraplegics Hospital (CEBA). For the Th8 contusion SCI model, young adult male Wistar rats (300–335 g, 12 weeks of age) were submitted to a moderate-severe contusion SCI. Briefly, rats were anesthetized with an intraperitoneal injection of sodium pentobarbital (45 mg/kg, Normon Veterinary Division) and Xilagesic (2% xylacine, 10 mg/kg, Calier). Once the absence of reflexes was confirmed, the rats were injected with a low dose of atropine (50 μg/kg bw; Brown Medical). After removing the Th8 vertebra, spinal cord contusion was performed with the IH impactor (Precision Systems and Instrumentation), applying a force of 200 kDyn without any additional dwell time. Postoperative care included a subcutaneous injection of Buprex (buprenorphine, 0.05 mg/kg; Schering Plough) and a prophylactic sub-cutaneous antibiotic injection 1 h after the lesion and on the following day (Baytril, Enrofloxacine, 1 mg/kg; Bayer). Injured rats received daily subcutaneous injections of either natural progesterone (16 mg/kg/d, Sigma-Aldrich, SCI+Prog. group) or vehicle (Castor oil, Sigma-Aldrich, SCI group) for 60 d until killing. The first injection was given to awake animals 1 h after injury.

##### Behavioral testing

Gait data were collected using CatWalk 7.1. The BBB scores (Basso et al., 1995) were additionally scored for coordination with the CatWalk RI (Koopmans et al., 2005), named RI-controlled BBB score. The rats with BBB scores of 9 or higher were further examined weekly by CatWalk 7.1 analyses. The number of rats and runs included in the data collection are listed in Table 3.

**Table 3:** Number of animals and CatWalk runs of Study 2

Study 2		#Animals	#Runs
Uninjured		5	18
Vehicle (SCI)	7 dpi	4	16
	30 dpi	7	32
	60 dpi	7	32
Experiment (SCI + Prog.)	7 dpi	7	27
	30 dpi	8	29
	60 dpi	7	27

Prog: progesterone.

#### Study 3

##### Th8 contusion SCI model

In this yet unpublished study, motor recovery after traumatic mid-thoracic SCI in three-month old female Fischer 344 rats (Charles River Deutschland GmbH; 160.1 ± 7.1 g) was compared with rats of 20–24 months of age (273.7 ± 23.7 g). Rats underwent a laminectomy at Th8 vertebral level followed by a 200 kDyn contusion generated by an IH Impactor (Precision Systems and Instrumentation) without additional dwell time. For surgical purposes, rats were under general inhalative anesthesia using a SomnoSuit unit (Kent Scientific) with 2% isoflurane/O_2_ gas mix. Analgesia was provided by subcutaneous injection of 0.05 mg/kg bw buprenorphine (Bupaq, Richter Pharma). Heart rate and oxygen saturation were monitored (SomnoSuit) and body temperature was maintained by a sensor-driven heating pad (Harvard Apparatus). Postoperative care included subcutaneous injection of 0.03 mg/kg bw buprenorphine twice daily for 2 d, 1–2 mg/kg bw meloxicam (Metacam; Richter Pharma) and 10 mg/kg bw Baytril (Bayer Pharma) daily for 7 d. The bladder was manually voided twice daily and once daily after reflexive bladder voiding was established (∼14 d).

Rats were housed in groups of 5 in a 12/12 h light/dark cycle and had food and water *ad libitum*. Experimental procedures were authorized by the ethical committee of the “Land Salzburg” (20910-TVG-79/17-2014) according to the European Directive 2010/63/EU on the protection of animals used for scientific purposes.

##### Behavioral testing

BBB tests were performed on days 1, 4, and 7 postinjury and weekly thereafter. The rats with BBB scores of 11 or higher were further examined weekly by CatWalk XT analyses. For the CatWalk XT analyses, rats were trained on the CatWalk device for at least two weeks (two times per week) before surgery and a baseline was created. Rats did not receive food rewards during the testing. The CatWalk data acquisition ended after 43 d for the young and 29 d for the aged group. The number of animals and runs included in the data collection are listed in Table 4.

**Table 4:** Number of animals and CatWalk runs of Study 3

Study 3		#Animals	#Runs
Young: injury at 3 months	0 dpi (uninjured)	19	91
	15 dpi	2	7
	22 dpi	6	29
	29 dpi	6	28
	36 dpi	6	25
	43 dpi	7	28
Old: injury at 20–24 months	0 dpi (uninjured)	12	131
	15 dpi	3	37
	22 dpi	6	40
	29 dpi	6	33

#### Study 4

This unpublished work was designed to study the effect of various undisclosed compounds on the recovery of locomotion following SCI. For the Th9 dorsal hemisection Scouten wire-knife transection SCI model, adult female Wistar rats (M&B Breeding) weighing 220–250 g at the time of operation were anesthetized with isoflurane (Forene, Abbott; 2–3% in O_2_ and N_2_O at a ratio of 1:2). Following laminectomy at Th8/9, the dura mater was opened at Th8/9 with a longitudinal cut and a dorsal hemisection injury was performed with a Scouten wire knife (Bilaney). After suture of the dura mater, a polyurethane rat intradural catheter (32 G, ReCathCo) was inserted at Th11 and epidurally guided to the lesion site. The catheter was filled with vehicle solution before it was inserted into the subarachnoid space in close proximity to the dura suture. Subsequently, it was connected to a prefilled osmotic minipump (Alzet pump model 2002), which was placed subcutaneously. Finally, the lesion area was covered with a piece of Nescofilm (Carl Roth), and overlying muscle and skin were sutured in layers. Immediately after surgery, animals received subcutaneous injections of 5 ml of physiological saline and 5 mg/kg carprofen (Rimadyl, Pfizer). Individual caging was provided until the animal had fully recovered from anesthesia. Postoperative care included manual bladder expression until normal bladder function returned, prophylactic antibiotic treatment (Baytril; Bayer Healthcare) for one week, and pain relief (Rimadyl; Pfizer) for 2 d postlesion. After a two-week infusion period, the osmotic minipump was removed under short anesthesia. During all surgical procedures, animals were placed on a heating pad to maintain body temperature. Institutional guidelines for animal safety and comfort were adhered to, and all surgical interventions and presurgical and postsurgical animal care were provided in compliance with the German Animal Protection law (State Office, Environmental and Consumer Protection of North Rhine-Westphalia, LANUV NRW, AZ 8.87-50.10.34.08.061). Experimental animals were housed in groups under standard conditions. Water and food were available *ad libitum*.

##### Behavioral testing

Four weeks before surgery, all animals were familiarized and pretrained in the behavioral tests. The overall HL function was assessed in an open field using the BBB score. Freely exploring rats were observed by two blinded examiners at 30 and 60 d postlesion and their HL movements were rated according to the BBB open field score. Differences in walking patterns were investigated using the CatWalk XT. The number of animals and runs involved in the CatWalk test are shown in Table 5. Food rewards were used throughout the entire study, and animals were trained to cross the horizontal glass runway without interruption.

**Table 5:** Number of animals and CatWalk runs of Study 4

Study 4	#Animals	#Runs
0 dpi [baseline (uninjured)]	19	63
30 dpi	16	42
60 dpi	12	36

##### Exclusion criteria

Some animals showed signs of automutilation. In the case of severe automutilation, the respective animal was killed prematurely. If only minimal signs of automutilation were detected, the respective animal was not included in the behavioral tests at individual test time points.

#### Study 5

This study was performed as published previously (Bieler et al., 2018) and designed to study the motor deficits following a cervical cortical spinal tract SCI. For the C4 bilateral dorsal column tungsten wire-knife transection SCI model, this study was conducted on female Fischer 344 rats (169 ± 8 g bw). Experiments were performed in accordance with the Directive 2010/63/EU of the European Parliament and of the Council and were approved by the local animal health commission (Land Salzburg 20 901-TVG-65/8-2013). Rats were purchased from Charles River Laboratories and were housed in groups of five in standard conditions, i.e., a 12/12 h light/dark cycle, and food and water were provided *ad libitum*.

The lesion rat group underwent a bilateral transection of the dorsal column at the fourth cervical segment. Before surgery, rats were deeply anesthetized using an intramuscular injection of 46.5 mg/kg bw ketamine (Narketan 10%, Vétoquinol), 2.3 mg/kg bw xylazine (Rompun, Bayer Austria GmbH) and 0.46 mg/kg bw acepromazine (Vanastress, Vana GmbH). Rats were placed on a homeostatic heating pad with body temperature monitoring via a rectal sensor to prevent hypothermia. The dorsal spine of the rat was exposed and a laminectomy at C4 was performed to expose the spinal cord. Using a blunt tungsten wire-knife device (M122, David Kopf Instruments), the dorsal column was precisely transected bilaterally (2.5-mm width, 1.1-mm depth) as previously described (Weidner et al., 1999, 2001; Sandner et al., 2013; Bieler et al., 2018). To prevent infections after surgery, rats were treated with 10 mg/kg bw Enrofloxacin (Baytril, 2.5% injection solution, Bayer Austria GmbH) daily for five consecutive days starting peri-operatively. Analgesia was provided by daily subcutaneous administration of 1.25 mg/kg bw Meloxicam (Metacam, Boehringer Ingelheim Vetmedica GmbH) for five consecutive days after surgery. In the first 2 d after surgery, 0.02 mg/kg bw buprenorphine (Bupaq, Richter Pharma AG) was injected subcutaneously twice a day. Upon signs of dehydration, 1–2 ml of 0.9% NaCl solution was injected subcutaneously. In this SCI model, bladder function is sufficient and did not require manual voiding.

##### Behavioral testing

Differences in gait parameters were investigated using the CatWalk XT. Two weeks before injury, rats were familiarized with the device and testing conditions. Rats crossed the CatWalk voluntarily without the use of food rewards. Rats were tested 30 dpi. The number of animals and runs involved in this study are shown in Table 6.

**Table 6:** Number of animals and CatWalk runs of Study 5

Study 5		#Animals	#Runs
C4	0 dpi (uninjured)	6	36
	30 dpi	6	36

### Establishment of the CatWalk parameter linear combination

The parameter linear combination was built on gait parameters extracted from the recorded videos by the CatWalk software. Only the gait parameters available in both the CatWalk 7.1. and CatWalk XT were considered for this study. Gait parameters from manually validated labeled runs were exported as run statistics. From 317 CatWalk XT parameters and 228 CatWalk 7.1. collected parameters, we included gait parameters related to mean values of paw statistics (60 parameters), step sequence (RI and sequence AB), BOS (two parameters), print position (two parameters), and number of paws supporting the walk ([Support_One + 2 × Support_Diagonal + 2 × Support_Girdle + 2 × Support_Lateral + 3 × Support_Three + 4 × Support_Four]/100). However, parameters directly related to green-pixel-intensity (ranged 0–255 in pixel-intensity units) were not used as they are often too sensitive to the experimental setting and walkway moisture. This intensity is closely related to paw pressure or weight support (Hamers et al., 2006). However, this exception does not include parameters that measure time (s) duration when max contact was realized. Gait parameters from the right and left paws were averaged except for body speed, which was averaged from all four paws, as performed in a recent PCA of CatWalk data from mice (Zimprich et al., 2018). This resulted in 22 parameters for consideration of ranked gait parameters by *t* test.

In order to develop a comprehensive measure which would efficiently examine the gait recovery differences between experimental and vehicle-treated rat groups, we had to first designate which gait parameters are the most descriptive of a spinal cord injured animal. To do this, we grouped uninjured rats from Studies 1 and 2 as controls. Our SCI group was pooled from vehicle moderate-severe SCI rats from Study 1 along with the vehicle SCI rats (60 d post-SCI) from Study 2. Next, we ranked gait parameters with a *t* test without assuming equal variances between these two groups for all individual gait parameters. To perform this *t* test, parameters from all runs of each rat were averaged. All gait parameters having *p *<* *0.01 from the *t* test were used to build the linear combination of CatWalk parameters. Of the parameters that reached our defined threshold, the effect size examined by Cohen’s *d* was found to be >1.3, which is considered to be a very large effect size (Sullivan and Feinn, 2012).

The linear combination of CatWalk parameters *p_LDA_* can be expressed as Equation 1, where *p_i_* is the *i*-th CatWalk gait parameter, *w_1,i_* is the corresponding parameter-weight, and *N_p_* is the number of gait parameters.

(1) $$p_{LDA}=\overset{N_{p}}{\underset{i=1}{\sum }}w_{1,i}p_{i}.$$

The parameter-weights *w_1,i_* of the linear combination were computed based on a machine learning method, namely LDA, which aims to maximize the between-class scatter and minimize the within-class scatter. Here, the LDA used gait parameter values from all runs. The method starts with calculating the within-class scatter matrix *S_w_* and between-class scatter matrix *S_b_* as shown in Equations 2, 3, where $x_{i}^{j}$ is the *i*-th run data of group *j*, $\mu_{j}$ is the mean of group *j*, *c *=* *2 is the number of group, *N_j_* is the number of run data in class *j*, and μ represents the mean of all classes.

(2) $$S_{w}=\overset{c}{\underset{j=1}{\sum }}\overset{N_{j}}{\underset{i=1}{\sum }}\left(x_{i}^{j}-\mu_{j}\right){\left(x_{i}^{j}-\mu_{j}\right)}^{T}$$

(3) $$S_{b}=\overset{c}{\underset{j=1}{\sum }}\left(\mu_{j}-\mu \right){\left(\mu_{j}-\mu \right)}^{T}.$$

To maximize the between-class scatter and minimize the within-class scatter, an eigen value problem as shown in Equation 4 was defined, where **w** is the eigen vector and λ is the eigen value.

(4) $$S_{w}^{-1}S_{b}w=\lambda w.$$

Here, we took the first eigen vector **w**_1_, which has the highest eigen value, and project our data to this eigen vector as given in Equation 5.

(5) $$p_{LDA}=w_{1}\cdot p=\overset{N_{p}}{\underset{i=1}{\sum }}w_{1,i}p_{i}.$$

### Analysis

Here, the *p_LDA_* parameter combinations were calculated for all run data from Studies 1 and 2 (total 107 run data, 57 run data from Study 1 and 50 run data from Study 2, total 25 animals, 4.28 ± 1.28 runs per animal). Thereafter, the differences between groups in each study were analyzed based on the resulting parameter combination *p_LDA_* from the average run data of each animal. For relative locomotor comparison, a statistical analysis of the thoracic SCI rats’ BBB score was also performed.

The statistical analyses between two groups were performed by *t* tests without assuming equal variances. The differences between three or more groups were investigated by ANOVA and multiple comparison tests with Bonferroni correction provided in MATLAB R2015a (8.5.0.). Groups were considered significantly different when the *p* values were below 0.05 (*p *<* *0.05).

## Results

The purpose of this study was to develop a combination parameter for CatWalk to be more predictive of gait recovery after SCI than any single CatWalk parameter considered alone or even more than the widespread BBB score (see Materials and Methods and below). Subsequently this parameter combination (built from Studies 1 and 2) was applied and validated in the assessment of gait recovery in the various experimental SCI paradigms shown here.

### CatWalk parameter linear combination

The gait parameters differing between control and SCI rats from Studies 1 and 2 with a *p *<* *0.01 from the *t* test without assuming equal variances (effect size, Cohen’s *d* > 1.3), as well as the values of the first eigen vector **w**_1_, are listed in Table 7. Although these SCI models directly affect HL function, the list of parameters contains several forepaw-related gait parameters. This confirms that FLs play an important and reinforced role after an SCI for the support and balance of bw during walking (Ghosh et al., 2010; Wilcox et al., 2017).

**Table 7:** List of gait parameters *p_i_* (*N_p_* = 9) and their corresponding parameter-weight *w_1,i_* for the linear combination of parameters

*w_1,i_*	*p_i_*
1	Forepaw swing time (s)
0.0015	Forepaw stride length (cm)
0.0005	Forepaw duty cycle (%)
−0.0103	Hindpaw BOS (cm)
0.00002	RI (%)
0.001	Body speed (cm/s)
−0.0001	AB sequence (%)
−0.0015	Forepaw max contact at (%)
−0.0017	Hindpaw stride length (cm)

AB sequence: Alternate (LF-RH-RF-LH).

RF: right forepaw; LF: left forepaw; RH: right hindpaw; LH: left hindpaw.

The gait parameter combination, $p_{LDA}=\overset{N_{p}}{\underset{i=1}{\sum }}w_{1,i}p_{i}$, obtained by a machine-learning LDA-based computational method described above (see Establishment of the CatWalk parameter linear combination), aims to maximize the between-class scatter and minimize the within-class scatter of data collected from Studies 1 and 2. A machine learning method that generates a simple linear combination of parameters was chosen for ease of use. In applications of data classification, LDA performs optimally in normally distributed data (Liong and Foo, 2013). Therefore, normality tests were performed.

Normality tests using the Shapiro–Wilk *W* test (using R version 3.5.1) were done on the run data for these parameters (Studies 1 and 2 combined). From the nine parameters, the normality tests were run separately for uninjured and injured groups for the seven parameters. The normality test was not relevant for two parameters, RI (%) and sequence AB (%). All RI (%) values from the uninjured group were at their maximum value of 100% (equal to a normal distribution with a variance of zero). From this uninjured group, 80% of the sequence AB (%) values were at their maximum value of 100%. From the injured group, around 30% of the data shown at their maximum value of 100%. Therefore, the distributions resemble one-sided normal distributions. From the 14 normality tests, 10 parameters show no difference from a normal distribution with *W*_Shapiro–Wilk_ > 0.95, and four parameters show similarity with a normal distribution with *W*_Shapiro–Wilk_ > 0.84. These four parameters only show similarity with a normal distribution because of the combination of two studies involving characteristic differences of rat strains used.

Here, the LDA was used to generate a linear combination of gait parameters based on the resulting first eigen vector, *p*_LDA_. The LDA was not used to classify data nor to statistically analyze data. Therefore, we considered the similarity with the normal distribution (*W*_Shapiro–Wilk_ > 0.84) adequate for this objective. RI (%) and sequence AB (%) have been shown as important parameters in several studies (Hamers et al., 2006; Šedý et al., 2008; Datto et al., 2015; Crowley et al., 2018); therefore, we considered that their distributions would not hinder the efficacy of generating a linear combination of gait parameters. The gait parameters are interdependent on each other and some weights act to balance the influence of other gait parameters. The resulting parameter combination LDA was used to assess the progression of gait recovery in all following studies presented here. An example of the *p*_LDA_ calculation can be found at https://github.com/mad-lab-fau/GaitParamCombSCI.

Specific definitions of the given parameters included within the *p*_LDA_ are listed below:

- Swing time (s) is calculated by the duration of non-contact with the walkway of a specific paw.
- Stride length (cm) is calculated by the distance between the center points of two consecutive positions of the same paw.
- Duty cycle (%) is the ratio of stand time to step cycle (duty cycle = stand time/step cycle). Stand time (s) is calculated by the duration of contact with the walkway of a specific paw. Step cycle (s) is calculated by the duration of two consecutive initial contacts of a specific paw (step cycle = stand time + swing time).
- BOS (cm) is calculated by averaging the width on the y-axis between either forepaws or hindpaws (BOS forepaws = *y*_RF_ – *y*_LF_; BOS hindpaws = *y*_RH_ – *y*_LH_).
- RI (%) is the ratio of the number of NSSP times four and the total number of paw placement (PP; Koopmans et al., 2005); RI = NSSP × 4/PP. There are six patterns that are considered as NSSP (Cheng et al., 1997): cruciate (CA: RF–LF–RH–LH; CB: LF–RF–LH–RH), alternate (AA: RF–RH–LF–LH; AB: LF–RH–RF–LH), and rotary (RA: RF–LF–LH–RH; RB: LF–RF–RH–LH). RF: right forepaw; LF: left forepaw; RH: right hindpaw; LH: left hindpaw.
- Body speed (cm/s) is calculated from each paw by dividing the distance by the time of two consecutive initial contacts.
- Sequence AB (%) is the percentage of a specific LF–RH–RF–LH paw sequence.
- “Max contact at (%)” is the ratio of “max contact at (s)” to the stand time (s), where “max contact at (s)” is the time when maximum contact with the walkway is measured. This is calculated from each paw and related to the point in which the braking phase turns into the propulsion phase.

It should be noted that the ranked parameters used in our combination consist of spatial dimensions (stride length, BOS and speed), temporal dimensions (swing, duty cycle, speed and max contact at), interlimb dimensions (AB sequence) and variation of interlimb coordination (RI) that encompass gait. All of these parameters were previously reported to provide the most variance in CatWalk based on PCA in mouse phenotyping experiments (Zimprich et al., 2018).

**Figure 2.**
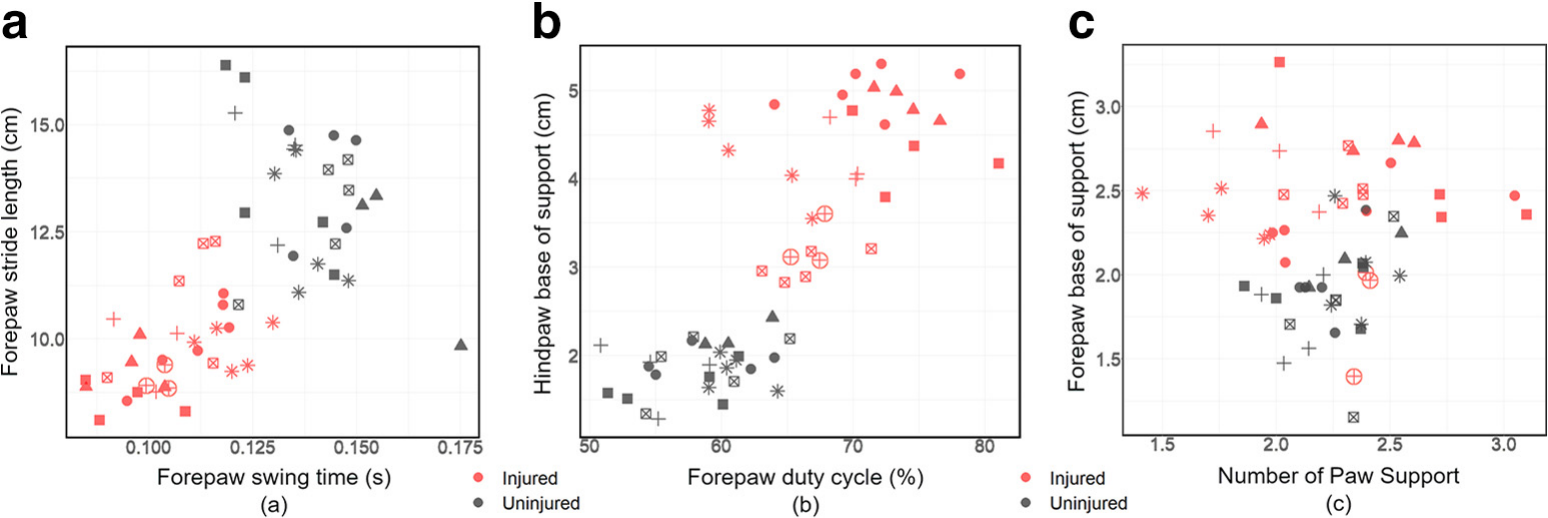
Scatter plot of several gait parameters obtained from the individual run data from Study 1. The injured (red) and uninjured (dark gray) animals are denoted by color here. The different shapes in each group represent the different individuals. ***a***, Forepaw swing time and forepaw stride length. ***b***, Forepaw duty cycle and hindpaw BOS. ***c***, Forepaw BOS and number of paw support = [Support_One + 2 × Support_Diagonal + 2 × Support_Girdle + 2 × Support_Lateral + 3 × Support_Three + 4 × Support_Four]/100.

The nine highest-ranked parameters between uninjured and injured groups cannot be represented in a single graph, however we show here coupled parameters that visualize the separation of injured and uninjured groups for Study 1 only. Figure 2 compares the two parameters (swing time and stride length) that were found to be significant in parameter ranking. Another two parameters (duty cycle and hindpaw BOS) are also shown in Figure 2, again depicting the separation of the uninjured and injured groups. Not all of the observed parameters depict differences. For example, two parameters (forepaw BOS and number of paw support) did not depict this separation, were not found to be significantly different and thus were not used (Fig. 2).

### Testing of the CatWalk parameter linear combination in distinct contusion SCI severities (Study 1)

**Figure 3.**
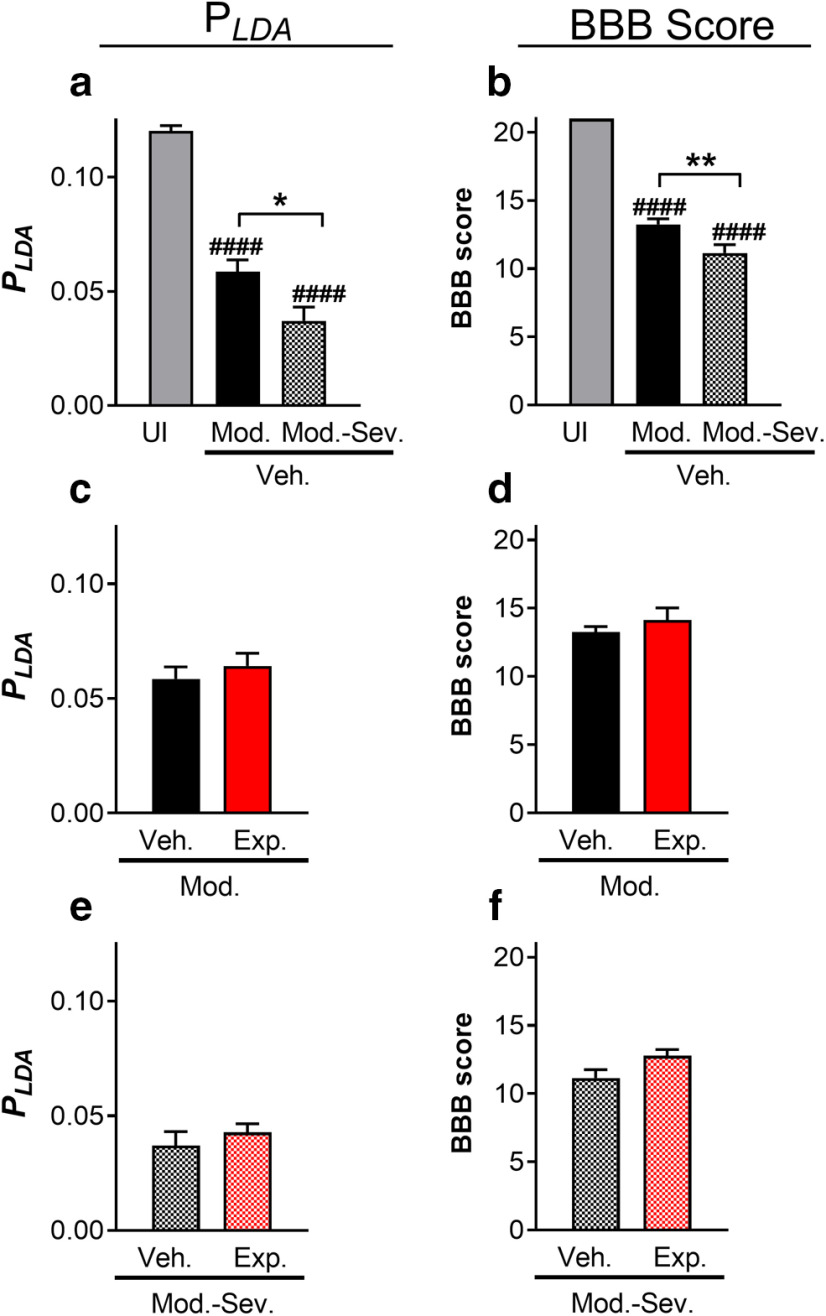
(***a***, ***b***) Combinations of gait parameters (*p_LDA_*) and BBB scores for each group in Study 1 without treatment (mean ± SEM). (***c***, ***d***) Treated and untreated pLDA and BBB scores for moderate contusions. (***e***, ***f)*** Treated and untreated pLDA and BBB scores for moderate-severe contusions. UI: uninjured; Veh.Mod.: vehicle moderate SCI; Veh.Mod-Sev.: vehicle moderate-severe SCI; Exp.Mod.: experimental moderate SCI; Exp.Mod-Sev.: experimental moderate severe SCI; Exp.Mod-Sev.: experimental moderate severe SCI; *p <* *0.05; **p <* *0.01; ####*p_compared with UI_* < 0.0001.

We tested the ability of the *p_LDA_* to distinguish differing lesion severities, as well as experimental treatment groups using the CatWalk data of Study 1 (Th8/9 moderate and moderate-severe contusion SCI). We observed that the uninjured rats had significantly higher values of *p_LDA_* (0.12) compared with the injured rats (60 dpi, *p_LDA_*0.03–0.06), as shown in Figure 3*A*. Furthermore, *p_LDA_* distinguished gait performance of the moderate (0.06) and moderate-severe (0.03) injured rats significantly. Treatment used in this study did not improve gait according to *p_LDA_*, as shown in Figure 3*C*,*E*. These results were similar to those obtained with the BBB scores (Fig. 3*B*,*D*,*F*). The values of *p_LDA_* and nine CatWalk parameters of vehicle moderate and vehicle moderate-severe groups are given in Table 8, as well as their *p* values calculated from the *t* test without assuming equal variances. It is important to note that most of the single CatWalk parameters did not display significant differences (Table 8), while the combination of those parameters *p_LDA_* was able to detect a significant gait difference.

**Table 8:** Mean and SD of *p*_LDA_ and several CatWalk parameters from vehicle moderate SCI and vehicle moderate-severe SCI models in Study 1 at 60 dpi and their *p* values as shown in Figure 3*A*

Study 1	Veh.Mod. Mean ± SD	Veh.Mod-Sev. Mean ± SD	*p* value
*p*_LDA_	0.059 ± 0.015	0.037 ± 0.016	0.018*
Forepaw swing time (s)	0.11 ± 0.01	0.11 ± 0.01	1
Forepaw stride length (cm)	10.5 ± 1.2	9.6 ± 0.8	0.21
Forepaw duty cycle (%)	64.7 ± 5.0	68.3 ± 3.4	0.25
Hindpaw BOS (cm)	3.6 ± 0.3	4.1 ± 0.7	0.15
RI (%)	93.8 ± 2.6	85.9 ± 10.5	0.077
Body speed (cm/s)	32.9 ± 6.5	27.5 ± 3.5	0.23
AB sequence (%)	64.1 ± 22.9	62.4 ± 16.8	1
Forepaw max contact at (%)	48.0 ± 3.9	52.9 ± 3.2	0.037*
Hindpaw stride length (cm)	12.2 ± 1.0	11.7 ± 1.6	1

*p <* *0.05.

### Examination of the CatWalk parameter linear combination across various studies with similar lesion type and severity (Studies 2 and 3)

**Figure 4.**
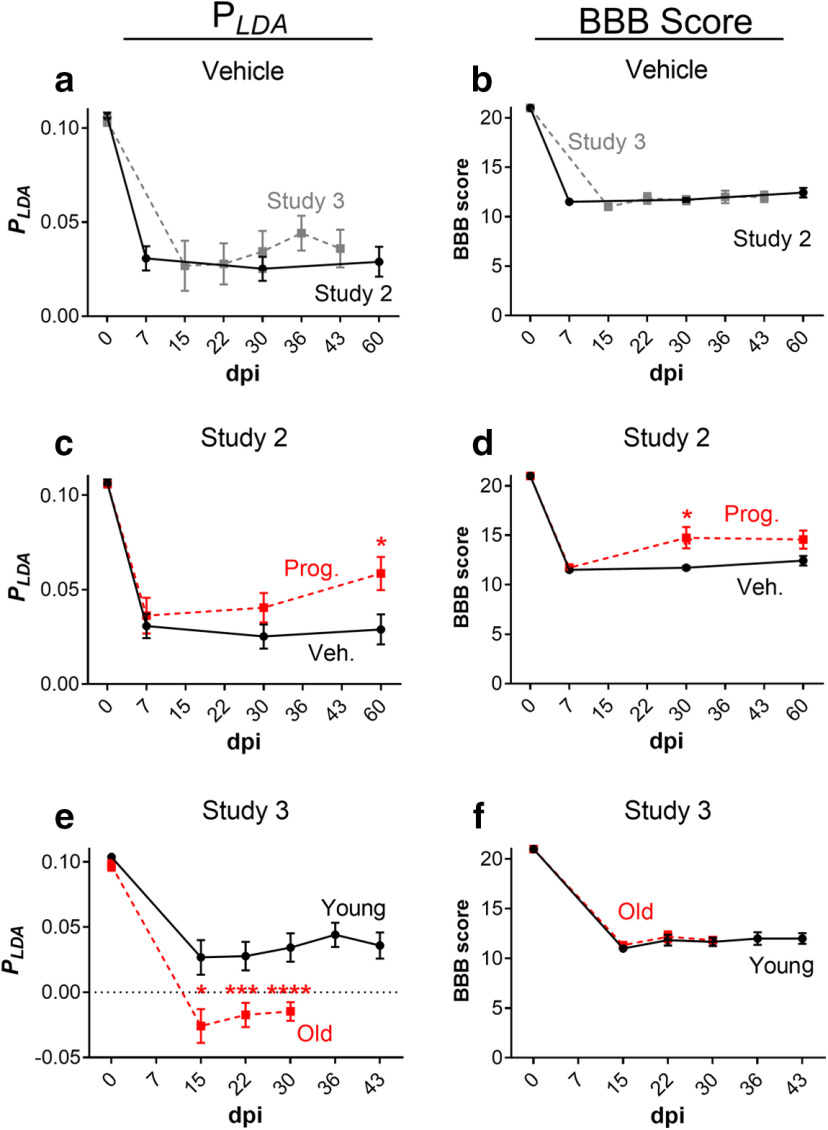
(***a***, ***b***) Combinations of gait parameters (*p_LDA_*) and BBB scores for vehicle and young groups in Studies 2 and 3, respectively (mean ± SEM). (***c***-***d***) The Two-way ANOVA in Study 2 shows significant effects on pLDA of both groups (*p * = 0.037) and time (*p *< 0.00001), without significant interaction. In the RI-controlled BBB score (Study 2), significant effects of both groups (*p * = 0.003) and time (*p *< 0.00001) were observed, as well as significant interaction (*p * = 0.049). (***e***-***f***) The Two-way ANOVA in Study 3 shows significant effect on pLDA of both group (*p *< 0.00001) and time (*p *< 0.00001), with significant interaction (*p *< 0.001). In the BBB score (Study 3), significant effect of time (*p *< 0.00001) was observed, but no significant effect of group and interaction; *p < 0.05, ***p < 0.001, ****p < 0.0001 Prog.: progesterone; Veh.: vehicle.

Following validation that our newly developed *p_LDA_* is capable of distinguishing thoracic SCI severity, we re-examined a previous SCI study (Study 2; Garcia-Ovejero et al., 2014) using a similar lesion type (i.e., thoracic contusion) and reporting differences in single CatWalk parameters between experimental treatment groups (hindpaw duty cycle, hindpaw swing, hindpaw BOS, phase dispersions, and RI). In this study, the regularity-index controlled BBB score also detected differences between the treatment groups. In addition, we also re-examined unpublished data from a study (Study 3) in which no significant differences in recovery were detected neither according to the BBB score nor in half of the single CatWalk parameters selected for the *p_LDA_*. First, we examined vehicle SCI rats from Study 2 (male Wistar rats) and Study 3 (female Fischer 344 rats), after Th8/9 contusion injury with a displacement over 1000 mm, which showed similar gait performance according to our *p_LDA_* (Fig. 4*A*). Correspondingly, the BBB scores of the vehicle SCI rats in these two studies showed that they were equally impaired and did not recover within a period of 60 d (Study 2), or 43 d (Study 3), respectively. Importantly, it should be noted that the use of different CatWalk systems (i.e., CatWalk 7.1 and CatWalk XT) for these two studies did not influence the comparability of data acquired here.

Examination of the *p_LDA_* and BBB scores from Study 2 (Fig. 4*C*,*D*) confirmed that treatment with natural progesterone improved gait performance, as previously reported (Garcia-Ovejero et al., 2014). Differences in gait performance between vehicle and treated rats were observed to be significant at 60 dpi with the *p_LDA_* and at 30 and 60 dpi with the RI-controlled BBB score. The values of *p_LDA_* and nine single CatWalk parameters for the SCI and SCI+PROG at 60 dpi are shown in Table 9, as well as their *p* values calculated from *t* tests without assuming equal variances.

**Table 9:** Mean and SD of *p*_LDA_ and several CatWalk parameters from the SCI and SCI+PROG models in Study 2 at 60 dpi and their *p* values as shown in Figure 4*C*

Study 2	SCI Mean ± SD	SCI+PROG Mean ± SD	*p* value
*p*_LDA_	0.029 ± 0.021	0.059 ± 0.027	0.040*
Forepaw swing time (s)	0.09 ± 0.01	0.12 ± 0.02	0.040*
Forepaw stride length (cm)	9.0 ± 1.5	10.4 ± 1.6	0.103
Forepaw duty cycle (%)	72.2 ± 3.2	67.6 ± 2.0	0.010*
Hindpaw BOS (cm)	5.1 ± 0.8	4.6 ± 0.8	0.28
RI (%)	89.8 ± 7.3	97.8 ± 2.4	0.027*
Body speed (cm/s)	27.9 ± 6.7	30.5 ± 8.1	0.52
AB sequence (%)	55.6 ± 15.4	66.3 ± 19.5	0.28
Forepaw max contact at (%)	43.5 ± 3.9	43.9 ± 1.7	0.79
Hindpaw stride length (cm)	11.6 ± 1.9	12.1 ± 1.2	0.60

*p <* *0.05

In Study 3, calculation of *p_LDA_* revealed a difference in gait recovery between rats undergoing contusion SCI at a young age (three months) versus at an old age (20–24 months; Fig. 4*E*). In contrast, no differences in the BBB score were detected between the two age groups (Fig. 4*F*). It should be noted that the young and old rats started at similar *p_LDA_* scores before injury, regardless of the differences in age and bodyweight at the given time. The values of *p_LDA_* and nine single CatWalk parameters of the young and old groups at 29 dpi are shown in Table 10, as well as their *p* values calculated from *t* tests without assuming equal variances.

### Comparison of the CatWalk parameter linear combination between distinct SCI lesion models (Studies 2, 4, and 5)

**Figure 5.**
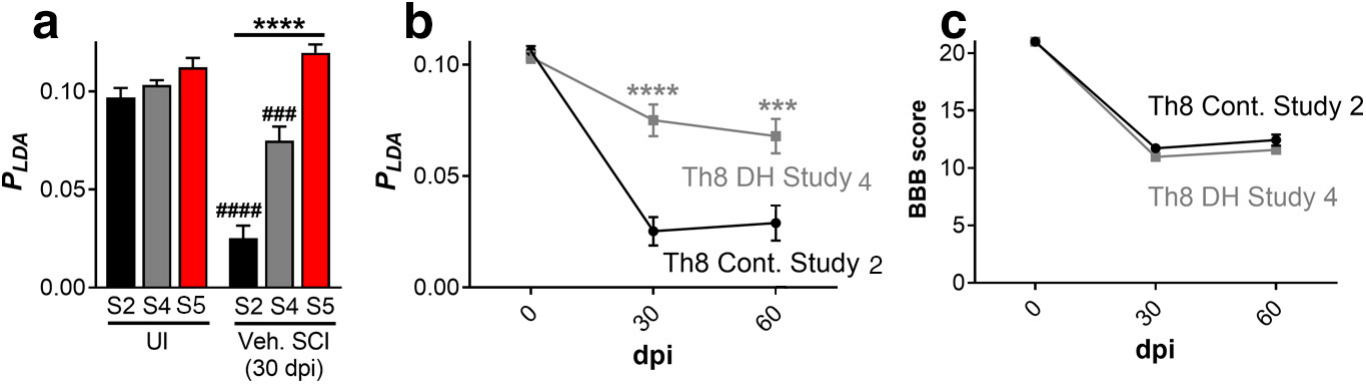
***a***, Combinations of gait parameters (*p_LDA_*) of uninjured rats and vehicle SCI rats in Study 2 (S2: Th8 contusion), Study 4 (S4: Th8/9 dorsal hemisection), and Study 5 (S5: C4 bilateral dorsal column lesion). ***b***, Combinations of gait parameters (*p_LDA_*) of vehicle SCI rats in Studies 2 and 4. ***c***, BBB scores of vehicle SCI rats in Studies 2 and 4; mean ± SEM; ***p <* *0.001, ****p <* *0.0001, ###*p_compared with UI_* < 0.001, ####*p_compared with UI_* < 0.0001; UI: uninjured; Veh.: vehicle; Cont.: contusion; DH.: dorsal hemisection.

Contusion injuries are the most common type of SCI experienced in patients, and thus this type of injury is commonly used in rodent SCI studies. However, several transection SCI models have been developed to examine the deficit brought on from injury to specific spinal tracts. Therefore, we challenged our new *p_LDA_* to distinguish various SCI lesion models, such as a bilateral dorsal column transection and a dorsal hemisection model, in comparison to the contusion SCI models described above. The results from the uninjured rats and vehicle SCI rats at 30 dpi acquired in Study 2 (Th8 contusion), Study 4 (Th8/9 dorsal hemisection), and Study 5 (C4 bilateral dorsal column wire knife lesion) are shown in Figure 5*A*.

In the rat thoracic contusion and dorsal hemisection SCI studies (Studies 2 and 4), the *p_LDA_* was able to distinguish the gait differences between uninjured and injured rats. It could also discriminate locomotor differences between the two SCI lesion models at 30 dpi. The values of the *p_LDA_* and nine single CatWalk parameters of the vehicle SCI animals from Studies 2 and 4 at 30 dpi are shown in Table 11, as well as their *p* value calculated from an ANOVA with a multiple comparison test by Bonferroni correction. The values of the *p_LDA_* and nine single CatWalk parameters of the uninjured and injured rats (30 dpi) from Study 5 are shown in Table 12. The single parameters obtained with the CatWalk system depicted different gait properties following C4 bilateral dorsal column lesion in rats (Study 5) compared with the Th8/9 dorsal hemisection (Study 4) or contusion SCI rats (Study 2). Among others, the injured rats in Study 5 showed significantly longer forepaw stride lengths compared with the uninjured rats, while the injured rats in Studies 1–4 mostly showed smaller forepaw stride lengths compared with the uninjured rats. Consequently, our parameter combination *p_LDA_* is not adequate to reflect the progression of gait recovery following C4 bilateral dorsal column transection in rats.

**Table 10:** Mean and SD of *p*_LDA_ and several CatWalk parameters from the young and old SCI models in Study 3 at 29 dpi and their *p* values as shown in Figure 4*E*

Study 3	Young Mean ± SD	Old Mean ± SD	*p* value
*p*_LDA_	0.034 ± 0.027	−0.015 ± 0.018	0.0049**
Forepaw swing time (s)	0.10 ± 0.01	0.088 ± 0.009	0.21
Forepaw stride length (cm)	11.0 ± 2.1	9.1 ± 1.1	0.079
Forepaw duty cycle (%)	67.4 ± 6.6	74.0 ± 3.1	0.061
Hindpaw BOS (cm)	4.9 ± 0.8	6.4 ± 0.7	0.0036**
RI (%)	87.0 ± 14.7	85.5 ± 5.4	0.82
Body speed (cm/s)	37.2 ± 9.7	26.4 ± 5.0	0.043*
AB sequence (%)	56.8 ± 20.3	43.1 ± 12.4	0.20
Forepaw max contact at (%)	48.6 ± 5.9	60.8 ± 4.4	0.0028**
Hindpaw stride length (cm)	13.5 ± 2.6	12.3 ± 1.1	0.33

*p <* *0.05, **p <* *0.01

**Table 11:** Mean and SD of *p*_LDA_ and several CatWalk parameters from Studies 2 and 4 at 30 dpi and their *p* values (ANOVA and multiple comparison tests by Bonferroni correction) shown in Figure 5*A*

30 dpi	Study 2 Mean ± SD	Study 4 Mean ± SD	*p* value
*p*_LDA_	0.025 ± 0.017	0.075 ± 0.029	<0.001***
Forepaw swing time (s)	0.09 ± 0.01	0.11 ± 0.02	0.006**
Forepaw stride length (cm)	9.2 ± 0.8	13.3 ± 2.9	0.001**
Forepaw duty cycle (%)	72.8 ± 3.1	58.8 ± 7.6	<0.0001****
Hindpaw BOS (cm)	5.0 ± 0.7	3.6 ± 0.9	0.003**
RI (%)	91.2 ± 2.8	78.8 ± 29.4	0.69
Body speed (cm/s)	29.3 ± 5.9	42.8 ± 10.6	0.007**
AB sequence (%)	44.2 ± 11.1	62.9 ± 28.9	0.26
Forepaw max contact at (%)	44.3 ± 5.7	41.3 ± 6.5	0.80
Hindpaw stride length (cm)	12.0 ± 1.7	16.8 ± 2.3	<0.0001****

**p <* *0.01, ***p <* *0.001, ****p <* *0.0001

**Table 12:** Mean and SD of *p*_LDA_ and several CatWalk parameters from Study 5 and their *p* values (*t* test without assuming equal variances) shown in Figure 5*A*

Study 5	Uninjured C4 Mean ± SD	C4 SCI, 30 dpi Mean ± SD	*p* value
*p*_LDA_	0.112 ± 0.012	0.120 ± 0.010	0.28
Forepaw swing time (s)	0.14 ± 0.01	0.14 ± 0.01	0.55
Forepaw stride length (cm)	13.1 ± 1.4	15.2 ± 0.6	0.01**
Forepaw duty cycle (%)	61.3 ± 4.1	57.9 ± 1.2	0.10
Hindpaw BOS (cm)	2.1 ± 0.1	2.0 ± 0.5	0.78
RI (%)	98.2 ± 1.6	99.4 ± 1.4	0.18
Body speed (cm/s)	32.6 ± 8.7	41.5 ± 3.9	0.06
AB sequence (%)	93.1 ± 11.1	83.3 ± 9.1	0.13
Forepaw max contact at (%)	43.1 ± 3.0	40.6 ± 2.9	0.18
Hindpaw stride length (cm)	12.9 ± 1.3	15.1 ± 0.6	0.008**

**p <* *0.01

To compare the progression of gait recovery in rats following different SCI types performed at the same spinal level, we compared the data acquired in Th8 contusion SCI (Study 2) with Th8 dorsal hemisection SCI [Study 4; *p_LDA_* (Fig. 5*B*) and BBB score (Fig. 5*C*)]. The *p_LDA_* revealed significant differences of walking function between respective injury models, displaying its enhanced sensitivity in differentiation. This was not detectable through BBB scoring. Again, we observed that despite the use of male and female Wistar rats in Studies 2 and 4, respectively, their *p_LDA_* before surgery were nearly identical, regardless of bodyweight and sex differences. It should be noted here that, similar to the bilateral dorsal column lesion used in Study 5, the dorsal hemisection performed in Study 4 also led to longer hindpaw stride lengths.

## Discussion

Sensitive tools to analyze gait recovery after SCI are necessary to better predict, from preclinical work, the potential effectiveness of treatments in clinical interventions. Gait represents a complex movement pattern, which can be divided into numerous movement components that can only be measured with a combination of single parameters which are dynamically interlinked. Current gait analyses predominantly rely on the BBB score, an observer-based open field locomotor scoring system which incorporates some static gait parameters from the HLs exclusively. Because of the non-linearity of this scoring system, ceiling effects at 8 and 13 points are often observed. A score of 8 has rhythmic movement of joints or plantar placement without weight support. A score of 13 has frequent to consistent weight supported plantar stepping with frequent FL–HL coordination, but the paw is rotated outwards. Moreover, it was found that experimental SCI targeting the dorsal region of the spinal cord has less influence on the BBB score (Schucht et al., 2002).

**Figure 6.**
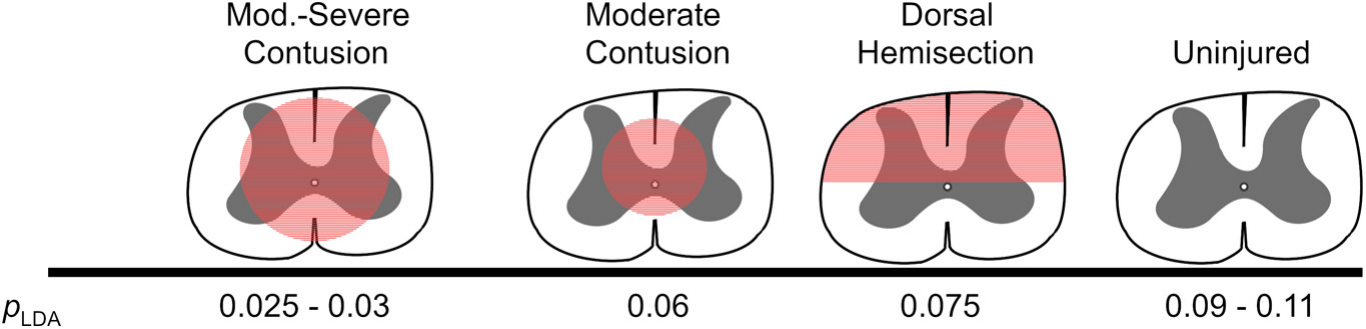
Schematic representation of the *p_LDA_* scores for varying lesion types and severities.

Here, we provide a novel unbiased combination of SCI-specific gait parameters acquired from the automated CatWalk gait analysis program, based on LDA (*p_LDA_*). It is noteworthy that our *p_LDA_* could be validated using parameters measured in the frame of five studies realized in various international laboratories, performed on different rat strains and from both sexes, by diverse experimenters using distinctive lesion types at different spinal levels. To the best of our knowledge, the robustness of this *p_LDA_* is unique for the analysis of CatWalk data in the field of preclinical SCI. The *p_LDA_* generated through the combination of nine SCI-related CatWalk static and dynamic gait parameters could reliably detect differences in gait performance, that researchers may have visually observed, although analysis of individual gait parameters or BBB score alone may not have disclosed differences to them. Although single CatWalk parameter analysis may be used to determine treatment effects following SCI, they lack the comprehensive analysis on the overall status of locomotion recovery as well as the comparison and standardization across studies currently used by the BBB score. Our *p_LDA_* indicates damage to the spinal cord involved in gross overground locomotor control by displaying a significant lowering of the overall value (Fig. 6). Thus, we believe this *p_LDA_* represents a significant improvement of our capacity and sensitivity to monitor the recovery of locomotion after SCI and the impact of therapeutic interventions.

Our study demonstrates that the comprehensive CatWalk-derived measure *p_LDA_* yields results, which are not affected by confounding factors such as sex, size and strain. This is not the case with individual CatWalk-derived single parameters, which have been shown to be influenced by sex, size and strain (Heglund et al., 1974; Taylor, 1978; Webb et al., 2003; Koopmans et al., 2007; Neckel et al., 2013; Jacobs et al., 2014; Machado et al., 2015; Neckel, 2015; Datto et al., 2016; Crowley et al., 2018). However, our goal with the *p_LDA_* approach was to add more power to our machine learning approach by neutralizing all these various sources of confounding variations. Therefore, data generated in studies performed in different laboratories on rats of different sex, strain, age, lesion type and lesion level were used to establish and validate the *p_LDA_*. In addition, it would be interesting to further examine the potential of our *p_LDA_* across species, e.g., in mice with similar thoracic SCI lesions.

It is intriguing that half of the nine parameters characterizing at best the uninjured and SCI rats were forepaw parameters. In addition to a simple functional adaption and weight transfer of HL to the FL (Wilcox et al., 2017), the neuroanatomical role played by propriospinal interneurons connecting the cervical and lumbar enlargements should also be considered (Ghosh et al., 2010; Flynn et al., 2011; Bareyre et al., 2004). These propriospinal connections mostly run along the ventrolateral tracts and are therefore more affected by a contusion injury than a dorsal hemisection. This could be appreciated through the drastic differences in the respective forepaw parameters following both lesions (Table 11, comparing Th8 Contusion, Study 2, vs Th8 dorsal hemisection, Study 4). In contrast, the BBB score is focused explicitly on HL alterations and may be why it is not as sensitive in distinguishing differences between these two SCI types (Fig. 5*C*).

In rodents, the reticulospinal and vestibulospinal tracts, located in the lateral to ventrolateral white matter, as well as the rubrospinal tracts, located in the dorsolateral white matter, are major elements of the locomotor control (Raineteau et al., 2002; Ballermann and Fouad, 2006; May et al., 2017; Asboth et al., 2018; Witts and Murray, 2019). Their preservation/disruption will be decisive in the locomotor parameters. White matter sparing was reported with progesterone treatment (Study 2) and forepaw parameters were directly affected. Further examination of direct sparing of ventrolateral descending tracts following progesterone treatment would be warranted. Moreover, plasticity of remaining spinal tracts has been reported to be crucial for locomotor recovery following SCI in rodents (Bareyre et al., 2004, 2005). Accordingly, our *p_LDA_* could detect the significantly different locomotor recovery following contusion in young and aged rats (Study 3), even in the presence of similar white matter sparing. It may be speculated that differences during recovery seen were age-related consequences in the capacity to undergo neuronal plasticity.

Our unbiased gait recovery analysis through the combination of SCI-specific CatWalk parameters relies heavily on the capacity of rats to retain or regain HL weight support (BBB score ≥10) required to transverse the runway. As a consequence, this *p_LDA_* method is not suited for the early phase of locomotor recovery during which weight support is not achieved, or in the case of very severe SCI. It should be stated, however, that this *p_LDA_* was specifically designed for thoracic lesions. When applied for the analysis of a bilateral dorsal column lesion at cervical level C4, the *p_LDA_* was unable to discern locomotor deficits compared with uninjured controls. We cannot rule out that other types of cervical lesions may be adequately characterized by our *p_LDA_* and this issue should be further examined.

*p_LDA_* represents a comprehensive linear behavioral measure reflecting the integrity/dysfunction of walking in SCI rats regardless of sex, strain and weight. Its strength is demonstrated in thoracic contusion SCI and defined partial transection models (dorsal hemisection). Moreover, certain conditions (progesterone treatment, young age) clearly improve gait function measure with *p_LDA_*. Although further work is necessary to fully understand the underlying mechanisms that lead to these differences. The fact that our *p_LDA_* integrates multidimensional parameters defining the quality of locomotion (spatial, temporal, interlimb and variation of interlimb coordination), as compared with the unidimensional and sequential BBB scoring system or single CatWalk parameters, explains its higher sensitivity. Therefore, we provide access to our gait recovery *p_LDA_*at https://github.com/mad-lab-fau/GaitParamCombSCI (simple downloadable Excel file) and hope that it will be used in the field of preclinical SCI for ease of comparisons in the recovery and quality of gait between various treatment groups.

## Synthesis

Reviewing Editor: Deanna Smith, University of South Carolina Decisions are customarily a result of the Reviewing Editor and the peer reviewers coming together and discussing their recommendations until a consensus is reached. When revisions are invited, a fact-based synthesis statement explaining their decision and outlining what is needed to prepare a revision will be listed below. The following reviewer(s) agreed to reveal their identity: Nick Jeffery, Mark Ballermann.

The reviewers and I are enthusiastic about the work and agree that this method of analyzing Catwalk data should allow better evaluation of differences in recovery between treatment and control groups and aid in identifying useful interventions. There was some concern that the authors need to check that their data sets are Gaussian, which is essential for LDA. It was also not clear whether the same weighting of parameters was applied to all data sets or whether a new calculation was used for each investigation. I have included the full comments to authors below, and it is important that you address each of the concerns before resubmission.

Reviewer 1: This is an interesting investigation and I greatly enjoyed reading it! I am unfamiliar with LDA, but i think I can follow your methods and the end results seem to be useful in the field of spinal cord injury research. I agree with you that an amalgamation of the (or, selected,) components of the Catwalk would be a great addition to the analytical methods we have available.

In addition, i would suggest some other general revisions:

a) The balance between the data that you show and its value in interpreting your study seems odd - there is a lot of information - though incomplete (see below) - on each study but much of this is not necessary for understanding the key part of this article - ie the amalgamation of the outcome measures.

b) At some stage there is a need to address the question of possible overfitting. I guess that you have partly addressed that through the use of this technique on several different studies (although it is slightly unclear to me whether the weighting used to derive the Plda was developed from study 1 and 2 and then used in all subsequent studies or whether you re-derived it for each study analysis).

c) your results suggest that this might be a useful technique for others to adopt but, unfortunately, i would guess that the uptake by other investigators might be impeded by a lack of access to appropriate statistical support. Is there a way that you might be able to make this more accessible to others?

I also have some comments about specific aspects of the design and analysis that appear below.

In addition - please could you include line numbers in future revisions? There is a need for some changes in English usage - but these are difficult to highlight without line numbers.

Specific comments

1. It is essential to test / state that the datasets used for generating the model had Gaussian distributions.

2. I think the use of t tests to determine which outcome variables to include in the LDA could be open to criticism. Partly, because you are relying on a small number of animals / datasets to generate this value and there is a risk that you select incorrectly because of random variation / multiplicity with this large number of t tests, and partly because the t test does not give a measure of magnitude of effect. The size of effect is in many ways more important than t test values.

3. The datasets used to select the outcomes for inclusion are not balanced well - there are different numbers of runs by each rat - meaning that specific rats are contributing more data than others.

4. It would help to state how many Catwalk outcomes (out of the ∼100 available) you considered for inclusion in the model.

5. Study 4: it is not clear where the catheter was placed - is this intradural (in the subarachnoid space) or intrathecal?

6. Please can you clarify that the model was built using the data from Studies 1 & 2 and then applied to all the other datasets? It seems to be what you have done but it is not stated overtly.

7. If this (6) is true, then perhaps the equation for the model can be reproduced with all the weightings stated so that it can be used by other laboratories?

8. How were the numbers of animals per study determined? The sample sizes are small, which might impair the accuracy of identification of appropriate outcome measures for inclusion in your model (see comments 2, 4 and 6).

Reviewer 2:

The authors describe a novel method of comparing measures of walking using the CatWalk apparatus following several spinal injuries in rats.

I think the empirical findings are presented well, for the most part. After more mild forms of thoracic damage, but not cervical injury, the linear discriminant analysis (LDA) supports the detection of deficits in walking among the treatment groups. This is consistent with the proposed wider use of the LDA approach in screening potential treatments for spinal cord injury.

Based on the weights reported in Table 7, most measures seem to contribute minimally aside from forepaw swing time. The authors discuss the probable explanation that hindlimb weight support is deficient and therefore forelimbs may adjust to compensate, along with other explanations.

I think the discussion would be strengthened by a clear description as to why pLDA differences would be sensitive and specific to treatment related improvements in walking when individual CatWalk measures; this may support the reader in deciding whether to apply the method described.

Minor

pg 5 - ‘From most of these studies, the gait recovery profile was reliably uncovered even when differences were not detectable by the BBB score alone’ - This sentence is imprecise. For which studies, and how are the measures more sensitive than the BBB score?

pg 22 - ‘Current gait analyses are predominantly relying’ suggest predominantly rely

pg 22 - ‘We provide a first of its kind unbiased combination...’ Probably the appropriate word would be ‘novel’.

pg 24 - ‘sparring’ should probably be sparing as written elsewhere.

Figure caption 2 - The individual points on the scatter plot should be described. Are the data presented grouped or individual runs across all animals? Inter- and intra-subject variability should be clear to the reader.
